# Gene variation and genetic differentiation among populations of the solitary mud dauber wasp *Trypoxylon* (*Trypargilum*) *albitarse* Fabricius 1804 (Hymenoptera, Crabronidae)

**DOI:** 10.1590/S1415-475738420150097

**Published:** 2015

**Authors:** Antonio C.B. Bergamaschi, Marco A. Del Lama

**Affiliations:** 1 Departamento de Biologia, Universidade Estadual do Centro-Oeste, Guarapuava, PR, Brazil; 2 Departamento de Genética e Evolução, Universidade Federal de São Carlos, São Carlos, SP, Brazil

**Keywords:** population genetics, wasps, Crabronidae, SSR markers

## Abstract

*Trypoxylon* is a genus of solitary crabronid wasps whose population genetics is poorly known. The purpose of the present study was to investigate the genetic variation and differentiation among five populations of *Trypoxylon albitarse*, a species widely distributed throughout the Neotropics, with records from Panama to northern Argentina. Eight species-specific microsatellite loci were used for genotyping 96 adult wasps (one female per nest) sampled at five sites in Brazil. The analysis of allelic richness and private alleles indicated high genetic diversity in the populations sampled. Pairwise comparisons using the *F*_*st*_ and *D*_*est*_ indices revealed significant differentiation for all, but one pair of populations. *F*_*st*_, *D*_*est*_, AMOVA and assignment test values pointed to inter-population differentiation. Additionally, the analysis of population structure using Bayesian and PCA methods characterized two alternative genetic groups. The Mantel test indicated no correlation between genetic and geographic distances. Despite evidence of considerable dispersal capacity for *T. albitarse*, the data indicate low to moderate population structuring in this species.

## Introduction

Among aculeate members of the order Hymenoptera, the family Crabronidae is a group of wasps that stands out for its worldwide distribution as well as for exhibiting a wide variety of nesting and foraging strategies. About 9,000 species have been described (Pulawski, 2014) and most do not exhibit social organization (Melo, 2000; O'Neill, 2001). Although some morphological studies have suggested that Crabronidae could form a monophyletic group, considered by some to be a sister group of Apidae (Lomholdt, 1982; Prentice, 1998; Melo, 1999; Michener, 2007), studies involving morphological (Alexander, 1992) and molecular data (Lohrmann, *et al*., 2008; Debevec *et al*., 2012) suggest that Crabronidae may not be a monophyletic clade and that bees may have arisen within this group. Despite having an important position in the Apoidea phylogeny, genetic studies involving crabronid wasps are scarce.

*Trypoxylon* is the most diverse genus of the family Crabronidae, with about 634 described species, which have a worldwide geographical distribution (Pulawski, 2014). Aspects of the natural history of these solitary hunter spider wasps are well known. However, the literature offers few reports addressing the population genetics of species of this genus or the other crabronid wasps using genetic markers. Until now, only allozyme markers were used to study the population genetics of *Trypoxylon* species; firstly, *Trypoxylon albitarse* and *Trypoxylon rogenhoferi* populations were studied by Peruquetti (2003) and thereafter, *Trypoxylon aurifrons, Trypoxylon nitidum* and *Trypoxylon lactitarse* populations by Santoni (2008). Despite the low level of heterozygosity of the markers employed, both authors found significant genetic differentiation among populations of these five species and suggested that this result would be due to a possible philopatry behavior of these wasps.

The solitary mud dauber *Trypoxylon albitarse* (Hymenoptera, Crabronidae) is widely distributed throughout the Neotropics, with records from Panama to northern Argentina, and is easily found on the walls of human constructions, such as buildings and bridges, especially if located near forested areas (Amarante, 1991, 2002). Based on the low recapture rates of marked specimens during a behavioral study, Amarante (1991) proposed that this species has a considerable high dispersal capacity. The wide geographical distribution of the species, the presumed high dispersal capacity and the successful nesting behavior in areas under anthropic pressure provide evidence of a lack of strong barriers to gene flow among populations of *T. albitarse*. However, the population genetic structure reported for the species (Peruquetti, 2003) and others of the same genus (Santoni, 2008) raises questions regarding the degree of dispersal among males and the effect of the presumed philopatric behavior of females (Melo, 2000) on the population genetic structure of species of *Trypoxylon*. Thus, the purpose of the present study was to estimate the degree of genetic differentiation among populations of *T. albitarse* using eight species-specific microsatellite loci.

## Material and Methods

### Sampling and fieldwork

**Figure 1:**
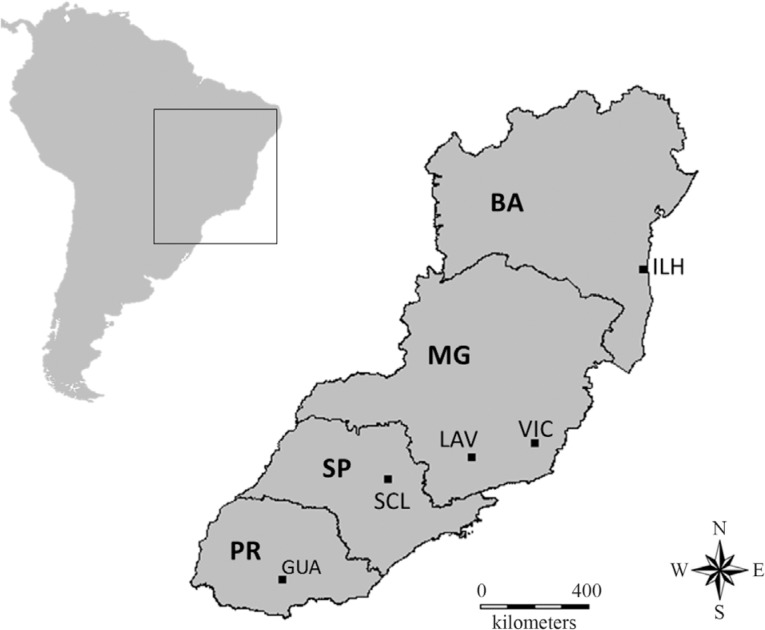
Map illustrating the geographical origin of five samples of *Trypoxylon albitarse* analyzed. The site codes were described in Table 1.

Specimens of *T. albitarse* were collected from five populations in Brazil: Ilhéus (state of Bahia), Viçosa (state of Minas Gerais), Lavras (state of Minas Gerais), São Carlos (state of São Paulo) and Guarapuava (state of Paraná) (Figure 1 and Table 1). The sampling sites were located in the northeastern, southeastern and southern regions of the country, with distances ranging from 238 km (Lavras to Viçosa) to 1797 km (Guarapuava to Ilhéus) between sites.

**Table 1:** Characterization of the sampling sites of *Trypoxylon albitarse* nests.

Code	Sampling site	Geographic coordinates
ILH	Ilhéus (BA), *Campus* da Universidade Estadual de Santa Cruz (UESC)	14°47′46″ S, 39°10′28″ W
VIC	Viçosa (MG), *Campus* da Universidade Federal de Viçosa (UFV)	20°45′14″ S, 42°52′53″ W
LAV	Lavras (MG), *Campus* da Universidade Federal de Lavras (UFLA)	21°13′40″ S, 44°57′50″ W
SCL	São Carlos (SP), *Campus* da Universidade Federal de São Carlos (UFSCar)	21°58′54″ S, 47°52′49″ W
GUA	Guarapuava (PR), Parque Municipal das Araucárias	25°20′44″ S, 51°27′31″ W

*Trypoxylon albitarse* nests with mud tubes found on the walls of human constructions were carefully opened with fine-tipped tweezers. When immature individuals in the pupal stage were found, the cocoons were placed in individual, duly labeled, plastic tubes, taken to the laboratory and kept at room temperature until the emergence of the adult stage, when the sex was determined, followed by storage of the material at −20 °C for subsequent DNA extraction.

### DNA extraction, microsatellite loci amplification and genotyping

Total DNA was extracted after the maceration of three pairs of legs or the mesosoma of adult specimens of *T. albitarse* using protocols based on either phenol-chloroform (Fernandes-Salomão *et al*., 2005) or Chelex 100 (Walsh *et al*., 1991). Samples were genotyped for nine species-specific microsatellite loci: *TALB01*, *TALB02*, *TALB03*, *TALB05*, *TALB06*, *TALB07*, *TALB09*, *TALB12* and *TALB14* (Almeida *et al*., 2013.). Polymerase chain reactions (PCR) were performed in an Eppendorf Mastercycler thermal cycler (Hamburg, Germany) using 250 μM of each dNTP, 2.5 mM of MgCl_2_, 0.5 μM of the fluorescently labeled forward primer, 0.5 mM of the reverse primer, 1x BioTools buffer and 1 U of *Taq* DNA polymerase (BioTools, Madrid, Spain) in a final volume of 10 μL. Amplification consisted of an initial denaturation step at 94 °C for 3 min, followed by 35 cycles of denaturation at 94 °C for 30 s, annealing of the primers at the specific temperature for each locus indicated by Almeida *et al*. (2013) for 20 s and chain extension at 72 °C for 1 min. Amplification cycles were followed by a final extension step at 70 °C for 10 min.

Approximately 13% of the samples were retyped to verify the reproducibility of the data and confirm the genotypes considered when weak or double peaks were found. To minimize the occurrence of missing data, PCR was performed up to three times in cases of the absence of amplification for a given locus.

The amplification products were analyzed after a run in the MegaBace-1000 automated sequencer (GE Healthcare, Buckinghamshire, United Kingdom). Fragment size was established by comparing the peaks in the set of samples to the ET550R size marker in the MegaBace Fragment Profiler program, version 1.2.

### Data analysis

As in most species of Hymenoptera, *T. albitarse* males develop from unfertilized eggs and are haploid, whereas females develop from fertilized eggs and are diploid. Based on this feature, only females were used in the genetic analysis. To avoid biased results due to the familial structure of the nests, only one female per nest was considered in the final dataset. The number of wasps analyzed per sampling site varied from 12 to 37 (Table 3).

Given the different sample size of each population of *T. albitarse* analyzed, the HP-Rare program, version1.1 (Kalinowski, 2005), which applies the rarefaction method (Kalinowski, 2004), was used to estimate allele richness and the occurrence of private alleles in each population. Genotype data were analyzed using the Arlequin program, version 3.11 (Excoffier *et al*., 2005) to obtain estimates of observed and expected heterozygosity for each locus and for the verification of linkage disequilibrium between loci. The significance of differences in genetic diversity between populations was tested using a paired *t*-test of arcsine transformed He values (Archie, 1985). Significant deviations from the Hardy-Weinberg equilibrium were determined using the GenAlEx program, version 6.5 (Peakall and Smouse, 2006). A 5% significance level (p < 0.05) was adopted for all statistical tests, and the sequential Bonferroni correction (Rice, 1989) was used when significant deviations were detected.

Population differentiation was estimated using total and pairwise *F*_*st*_ (Weir and Cockerham, 1984) and *D*_*est*_ (Jost, 2008) values using the FSTAT 2.9.3.2 (Goudet, 2001) and GenAlEx 6.5 (Peakall and Smouse, 2006) programs, respectively. In order to test the association of pairwise results obtained for both indexes, the Pearsons *r*-correlation coefficient was estimated using the program STATISTICA 7.0 (StatSoft Inc. 2004). Additionally, the partition of genetic variation within and among populations was estimated through Analysis of Molecular Variance (AMOVA) (Excoffier *et al*., 1992) using the program Arlequin 3.11 (Excoffier *et al*., 2005). The level of genetic differentiation among populations was also estimated by an assignment test conducted in the GenAlEx program, version 6.5 (Peakall and Smouse, 2006), which indicates the percentage of individuals correctly assigned to their original populations.

Population structure was investigated using a clustering approach with Bayesian inference in the Structure program, version 2.3.4 (Pritchard *et al*., 2000), for which no *a priori* information was provided on the origin of individuals. Using the mixed ancestry model with independent allele frequencies among populations, five simulations were conducted for each inferred *K* (number of genetic clusters), which ranged from 1 to 5. In each run, 1,000,000 repetitions of burn-in were conducted, followed by 5,000,000 MCMC repetitions. The estimated probabilities for each run were used to estimate Δ*K* (Evanno *et al*., 2005), which represents the most probable number of genetic clusters in the database.

Principal component analysis (PCA) was performed to identify the distribution of genetic variation in the geographic samples using the GenAlEx program (Peakall and Smouse, 2006). This program was also employed to test the correlation between genetic and geographic distances using a Mantel test (Mantel 1967).

The inbreeding coefficient (*F*_*is*_) (Weir and Cockerham, 1984) was verified using the program FSTAT 2.9.3.2 (Goudet, 2001). The software BOTTLENECK 1.2.02 (Piry *et al*., 1999) was used to test the heterozygosity excess and to estimate possible genetic bottlenecks signatures in all populations. As recommended by Piry *et al*. (1999) for microsatellite data, a two-phase mutation model (TPM) was assumed, with 95% single-step mutations and 5% multiple-step mutations, and variance among multiple steps of 12. Subsequently, heterozygosity excess significance over all loci (p) was determined by the Wilcoxon's test.

The Kingroup2 program (Konovalov *et al*., 2004) was used to determine kinship among females of each population through estimates of maximum likelihood using the approach described by Queller and Goodnight (1989) and Goodnight and Queller (1999).

## Results

### Genetic diversity

*Trypoxylon albitarse* females (n = 96) from the five sampling sites were genotyped for nine microsatellite loci. As locus *TALB12* was monomorphic in all samples, only eight loci were considered in the analysis. The lack of amplification for some loci, even after repetitions of the PCR, generated a missing data rate of nearly 3%.

Table 2 displays the estimates of allelic richness (A) and private alleles (Ap). Table 3 displays data on sample size, estimates of expected (He) and observed (Ho) intralocus and mean heterozygosity as well as significant deviations from the Hardy-Weinberg equilibrium. No linkage disequilibrium was observed between the pairs of loci used in the genetic analyses.

**Table 2:** Allelic richness (A) and private alleles (Ap) estimated using rarefaction method at eight microsatellite loci of five populations of *Trypoxylon albitarse*. The site codes were described in Table 1.

Locus	ILH		VIC		LAV		SCL		GUA	
	A	Ap	A	Ap	A	Ap	A	Ap	A	Ap
*TALB01*	2.87	0.26	2.29	0.49	3.65	1.08	1.65	0.20	2.05	0.006
*TALB02*	3.60	0.37	2.10	0.19	2.26	0.36	2.77	0.69	2.56	0.58
*TALB03*	3.15	0.01	3.42	0.10	3.09	0.01	3.34	0.09	3.40	0.51
*TALB05*	3.83	0.15	5.23	0.84	3.70	0.45	4.95	1.73	4.62	0.32
*TALB06*	2.90	0.0006	3.18	1.64	3.42	0.37	3.57	0.49	3.53	0.18
*TALB07*	5.20	0.71	5.90	1.26	6.35	2.18	5.20	1.12	6.27	2.37
*TALB09*	1.00	0	1.00	0	1.52	0.20	1.00	0	1.96	0.64
*TALB14*	1.65	0.23	1.88	0.56	1.00	0	1.97	0.4	1.66	0.46

**Table 3:** Sample size (n), observed (Ho) and expected (He) intralocus heterozygosity and chi-square values for deviations from Hardy-Weinberg Equilibrium (HWE) in five populations of *Trypoxylon albitarse* from Brazil genotyped at eight microsatellite loci. The site codes were described in Table 1.

Population		*TALB01*	*TALB02*	*TALB03*	*TALB05*	*TALB06*	*TALB07*	*TALB09*	*TALB14*	Mean
ILH (n = 17)	H_o_	0.41	0.31	0.58	0.29	0.25	0.64	-	0.17	0.38
	H_e_	0.57	0.71	0.7	0.74	0.67	0.86	-	0.16	0.63
	HWE	9.66	23.28*	3.44	33.88*	14.45*	86.19	-	0.15	
VIC (n = 16)	H_o_	0.12	0.43	0.87	0.57	0.42	0.73	-	0.12	0.46
	H_e_	0.41	0.36	0.69	0.87	0.62	0.91	-	0.23	0.58
	HWE	21.44*	1.25	4.11	41.27	4.9	53.81	-	16.08	
LAV (n = 14)	H_o_	0.14	0.28	0.61	0.57	0.58	0.78	0	-	0.42
	H_e_	0.72	0.37	0.64	0.72	0.72	0.93	0.14	-	0.6
	HWE	33.05*	14.46	5.07	6.78	12.91*	101.01	13*	-	
SCL (n = 37)	H_o_	0.13	0.51	0.56	0.86	0.64	0.89	-	0.32	0.56
	H_e_	0.17	0.55	0.66	0.84	0.7	0.86	-	0.28	0.58
	HWE	37.2	78.03	7.88	60.93	31.18*	46.44	-	1.26	
GUA (n = 12)	H_o_	0.16	0.16	0.66	0.75	0.66	0.58	0.09	0.08	0.39
	H_e_	0.3	0.47	0.65	0.84	0.73	0.92	0.25	0.16	0.54
	HWE	4.65	12.52	11	12.93	8.24	93.81*	11.03*	24*	

- Monomorphic loci.

* Significant deviation from HWE even after sequential Bonferroni correction (p < 0.05).

### Genetic structure

Estimates of differentiation using the *F*_*st*_ and *D*_*est*_ indices for pairs of populations were obtained for all loci. The results indicated significant genetic differentiation between all pairs of populations, but one (Lavras *vs*. Guarapuava) (Table 4). The Pearson's *r* correlation coefficient among the pairwise values of both indexes revealed a positive association between them (*r* = 0.994). Overall *F*_*st*_ and *D*_*est*_ values [0.11 (p < 0.01) and 0.13 (p < 0.01), respectively] indicated significant population differentiation. The distribution of genetic variation within and among populations was determined using AMOVA (Table 5). The assignment test indicated that 76% of individuals analyzed were properly assigned to their populations of origin.

**Table 4:** *F*_*st*_ (below the diagonal) and *D*_*est*_ (above the diagonal) values for pairs of *Trypoxylon albitarse* populations from five sites in Brazil genotyped at eight microsatellite loci. The site codes were described in Table 1.

	ILH	VIC	LAV	SCL	GUA
ILH	-	0.100	0.095	0.085	0.102
VIC	0.062	-	0.275	0.082	0.212
LAV	0.061	0.131	-	0.231	0.013*
SCL	0.049	0.050	0.110	-	0.165
GUA	0.064	0.108	0.032*	0.085	-

* No significant genetic differentiation (p < 0.05).

**Table 5:** Distribution of genetic variation within and among *Trypoxylon albitarse* populations genotyped at eight microsatellite loci according to AMOVA.

Source of variation	Sum of squares	Variance components	Percentage of variation	p
Among populations	40.98	0.23	12.28	0
Within populations	315.35	1.68	87.72	0

**Figure 2:**
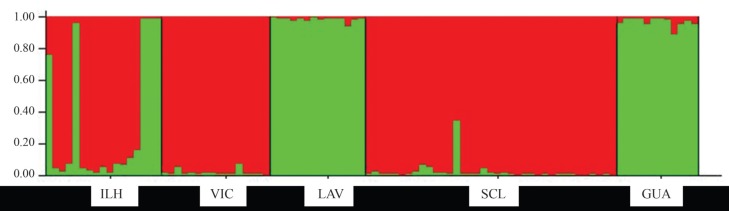
Bayesian clustering plots of *Trypoxylon albitarse* populations genotyped using eight microsatellite loci when *K* = 2 [each color represents one genetic cluster; each vertical column represents one individual and its chance of assignment to each of the two alternative genetic clusters (scale on the left)]. The site codes were described in Table 1.

**Figure 3:**
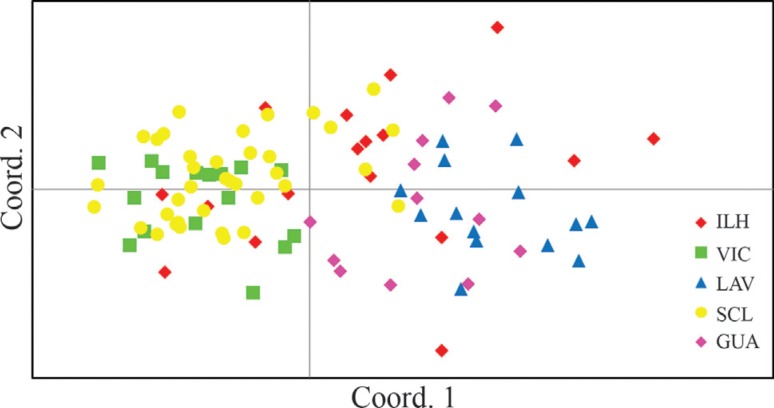
Principal component analysis based on genetic distances obtained by genotyping at eight microsatellite loci for the females from five populations of *Trypoxylon albitarse*. Axes 1 and 2 explain 30.1% and 18.4% of the genetic variation detected, respectively. The site codes were described in Table 1.

The differentiation pattern obtained through Bayesian analysis identified two alternative genetic clusters (*K* = 2) (Figure 2). A similar pattern was found using PCA (Figure 3). Both analyses revealed similar results and indicated that individuals from Ilhéus could be assigned to one or another of the two genetic groups identified in the analysis. The Mantel test indicated no correlation between genetic and geographic distances (*r* = −0.47; p = 0.1).

*F*_*is*_ values over all loci were positive and significantly different from zero in the five populations, ranging from 0.154 to 0.285 [0.157 (p < 0.01), 0.169 (p < 0.01), 0.285 (p < 0.01), 0.154 (p < 0.05) and 0.223 (p < 0.01) for Ilhéus, Viçosa, Lavras, São Carlos and Guarapuava respectively].

The test carried out in the program BOTTLENECK resulted in significant values, indicating a possible bottleneck effect for all the populations studied. The Wilcoxon sign-rank test for heterozygosity excess, using the two phase mutation model, resulted in the values 0.187 (p < 0.01), 0.632 (p < 0.01), 0.204 (p < 0.001), 0.187 (p < 0.01) and 0.101 (p < 0.01) for Ilhéus, Viçosa, Lavras, São Carlos and Guarapuava, respectively.

Although some pairwise estimates of relatedness (*r*) were close to 25% (rarely above this value), low mean relatedness values between pairs of females of each population were observed [Ilhéus (*r* = 0.002 ± 0.25), Lavras (*r* = 0.012 ± 0.22), Viçosa (*r* = 0.014 ± 0.3), São Carlos (*r* = −0.0007 ± 0.24) and Guarapuava (*r* = −0.003 ± 0.28)].

## Discussion

Differences among populations were detected regarding the degree of variation in the microsatellite loci. As an example, *TALB09* and *TALB14* loci exhibited polymorphism in two and four populations, respectively. Allelic richness per locus was estimated for the five populations and private alleles were detected in at least one locus for each population analyzed. According to Kalinowski (2005), the number of alleles or allelic richness is a simple measure of genetic diversity that is highly dependent on sample size. Thus, the rarefaction method (Kalinowski, 2004) was employed to estimate allelic richness and the number of private alleles and minimize the sample size effect. The analysis revealed high level of allelic richness at most loci in each population (Table 2), including Guarapuava, which was the population with the smallest sample (n = 12).

Expected intralocus heterozygosity did not differ substantially among populations, except for *TALB01* (0.17 to 0.72). Comparatively, the smallest variation in He among populations (0.14 to 0.25) occurred at locus *TALB09* and even the highly polymorphic locus *TALB07* exhibited a low level of variation (0.86 to 0.93). Thus, mean expected heterozygosity did not differ substantially among populations (range: 0.54 to 0.63) (Table 3), which was confirmed by a paired *t*-test using expected intralocus heterozygosity values for each population. Despite the similar heterozygosities, the number of private alleles at each locus differed (Table 2), suggesting inter-population differentiation.

The verification of Hardy-Weinberg equilibrium at each locus and *T. albitarse* population revealed that all loci, except *TALB03*, had at least a significant p-value (< 0.05). However following the Bonferroni correction, most deviations were found not to be significant. Deviations from Hardy-Weinberg equilibrium are generally associated with inbreeding, population structuring, the effects of natural selection, preferential mating, and/or the presence of null alleles (Templeton, 2011). Among these alternatives, the action of the natural selection seems unlikely, given that microsatellite markers are usually considered neutral and therefore free of the effects of this evolutionary mechanism. A high hit rate in the repeated genotyping (about 13% of the female wasps of this study) associated to a positive amplification of 174 haploid males of *T. albitarse* genotyped for the same loci of this study (Bergamaschi *et al*., in press) indicated that null alleles may not be common in these loci. High frequencies of mating between relatives (endogamy) would certainly generate deviations in the Hardy-Weinberg equilibrium at all loci and not only in isolated cases, as observed in the present study.

*D*_*est*_ values were higher than the corresponding *F*_*st*_ values and a positive association was found between the two indices (Table 4). Furthermore, AMOVA and the assignment test were also consistent with the *F*_*st*_ and *D*_*est*_ values, thereby confirming that the populations analyzed are not genetically homogeneous, but show a moderate differentiation level according to the criterion of Wright (1978).

Bayesian analysis and PCA pointed to similar results. Two alternative genetic clusters were identified: one composed by the Viçosa and São Carlos populations and the second by the Lavras and Guarapuava populations. Individuals from Ilhéus were distributed between the two alternative groups. As expected, the Mantel test revealed no correlation between genetic and geographic distances.

The absence of genetic homogeneity, associated to the significant values of the inbreeding coefficient (*F*_*is*_) among populations could be partially attributed to the presumed philopatry of *Trypoxylon* females. If an area is colonized by a small number of females, this results in a founder effect. If the growth of local populations occurs by the reproduction of the original nests, with the construction of satellite nests by daughters of the founding females, local populations would consist of few familial groups. Thus, despite the dispersal of males, some structuring would be possible due to the behavior of daughters nesting close to their native site. This hypothesis is in accordance with the possible recent demographic bottlenecks detected in all populations of *T. albitarse* studied. However, kinship analysis showed low genetic relatedness among females of the same population, suggesting that the dispersal ability of males may be sufficient to generate a low *r* values among females of the same population. It is worthy of note that this result is not due to the low resolution power of the genetic markers employed, as *r* values used to estimate the relatedness among individuals from the same nest were consistent with those expected for a predominantly monogamous genetic system (Bergamaschi *et al*., in press).

Little information is available on flight and dispersal capacity among species of *Trypoxylon*. Amarante (1991) reported a low recapture rate of adult specimens of *T. albitarse* during a behavioral study (4.6% and 15.1% for females and males, respectively) and reported that specimens marked during nesting activity were observed up to 42 days after marking. Based on these findings, the author proposed that this species has good dispersal capacity. Studies involving other species of the genus also suggest that these wasps have a high emigration rate (Freeman, 1981; Molumby, 1997; Buschini and Bergamaschi, 2010, 2014; Buschini and Donatti, 2012). However, evidence regarding the degree of dispersal in *T. albitarse* is not coherent with the inter-population genetic differentiation observed in the present investigation and the one by Peruquetti (2003).

In conclusion, this study makes a significant contribution to knowledge on the population genetics of cabronid wasps, which has been rarely studied. Given the relevance of these wasps to the phylogeny of bees and the present findings of moderate inter-population heterogeneity, further studies involving the genotyping of other populations of *T. albitarse* in a more limited geographical scale, as well as phylogenetically related species, are needed to obtain more conclusive results about the effects of gene flow and female philopatry on the genetic differentiation among populations of *Trypoxylon* species.
